# Neuroprotection and neuroregeneration of retinal ganglion cells after intravitreal carbon monoxide release

**DOI:** 10.1371/journal.pone.0188444

**Published:** 2017-11-27

**Authors:** Julia Stifter, Felix Ulbrich, Ulrich Goebel, Daniel Böhringer, Wolf Alexander Lagrèze, Julia Biermann

**Affiliations:** 1 Eye Center, Medical Center—University of Freiburg, Killianstrasse 5, Freiburg, Germany; 2 Faculty of Medicine, University of Freiburg, Freiburg, Germany; 3 Department of Anesthesiology and Intensive Care, Medical Center—University of Freiburg, Hugstetter Strasse 55, Freiburg, Germany; 4 Department of Ophthalmology, University of Muenster Medical Center, Domagkstrasse 15, Muenster, Germany; Universidade Federal do Rio de Janeiro, BRAZIL

## Abstract

**Purpose:**

Retinal ischemia induces apoptosis leading to neurodegeneration and vision impairment. Carbon monoxide (CO) in gaseous form showed cell-protective and anti-inflammatory effects after retinal ischemia-reperfusion-injury (IRI). These effects were also demonstrated for the intravenously administered CO-releasing molecule (CORM) ALF-186. This article summarizes the results of intravitreally released CO to assess its suitability as a neuroprotective and neuroregenerative agent.

**Methods:**

Water-soluble CORM ALF-186 (25 μg), PBS, or inactivated ALF (iALF) (all 5 μl) were intravitreally applied into the left eyes of rats directly after retinal IRI for 1 h. Their right eyes remained unaffected and were used for comparison. Retinal tissue was harvested 24 h after intervention to analyze mRNA or protein expression of Caspase-3, pERK1/2, p38, HSP70/90, NF-kappaB, AIF-1 (allograft inflammatory factor), TNF-α, and GAP-43. Densities of fluorogold-prelabeled retinal ganglion cells (RGC) were examined in flat-mounted retinae seven days after IRI and were expressed as mean/mm^2^. The ability of RGC to regenerate their axon was evaluated two and seven days after IRI using retinal explants in laminin-1-coated cultures. Immunohistochemistry was used to analyze the different cell types growing out of the retinal explants.

**Results:**

Compared to the RGC-density in the contralateral right eyes (2804±214 RGC/mm^2^; data are mean±SD), IRI+PBS injection resulted in a remarkable loss of RGC (1554±159 RGC/mm^2^), p<0.001. Intravitreally injected ALF-186 immediately after IRI provided RGC protection and reduced the extent of RGC-damage (IRI+PBS 1554±159 vs. IRI+ALF 2179±286, p<0.001). ALF-186 increased the IRI-mediated phosphorylation of MAP-kinase p38. Anti-apoptotic and anti-inflammatory effects were detectable as Caspase-3, NF-kappaB, TNF-α, and AIF-1 expression were significantly reduced after IRI+ALF in comparison to IRI+PBS or IRI+iALF. Gap-43 expression was significantly increased after IRI+ALF. iALF showed effects similar to PBS. The intrinsic regenerative potential of RGC-axons was induced to nearly identical levels after IRI and ALF or iALF-treatment under growth-permissive conditions, although RGC viability differed significantly in both groups. Intravitreal CO further increased the IRI-induced migration of GFAP-positive cells out of retinal explants and their transdifferentiation, which was detected by re-expression of beta-III tubulin and nestin.

**Conclusion:**

Intravitreal CORM ALF-186 protected RGC after IRI and stimulated their axons to regenerate in vitro. ALF conveyed anti-apoptotic, anti-inflammatory, and growth-associated signaling after IRI. CO’s role in neuroregeneration and its effect on retinal glial cells needs further investigation.

## Introduction

Retinal neurons, especially retinal ganglion cells (RGC), are highly susceptible to oxygen deprivation [1]. Ischemic or hypoxic conditions of the retina (e.g., retinal vascular occlusion, ischemic optic neuropathy, diabetic retinopathy) lead to neurodegeneration. Due to an increasing elderly population in many countries, the socioeconomic impact of visual impairment and blindness resulting from such diseases will increase in the future. An ischemia-reperfusion-injury (IRI) is thus the unifying pathophysiological process. The resulting neuronal damage is often irreversible due to reduced regenerative effectiveness.

It is well known that injured neurons and their glial environment are equipped with counteractive measures in cases of neurodegeneration [2] (e.g., upregulation of neurotrophic factors [3], activation of anti-apoptotic proteins and genes [4], and re-expression of growth-associated molecules [5–7]). However, the simultaneously induced apoptotic [8], inflammatory, and growth-inhibiting defenses ultimately prevail, leading to neurodegeneration, chronic microglia activation, and astrogliosis. Neuroprotective approaches should be multimodal and thus simultaneously address the currently known stressors involved in retinal neurodegeneration.

Carbon monoxide (CO) plays a crucial role in the central nervous system (CNS) for a host of functions [9, 10]. CO is an endogenously produced gasotransmitter originating primarily from heme metabolism. The upregulation of heme oxygenase-1 (HO-1) leading to CO production is another requisite of intrinsic neuroprotection to maintain cell homeostasis in the CNS [11, 12]. In the brain and retina, exogenously applied CO also mediates protection of neuronal tissue after ischemia and other neurodegenerative disorders [13–15]. Thus, pharmacological imitation, modulation, and amplification of CO signaling represent promising therapeutic strategies for general nervous system and ophthalmological disorders.

CO has shown cell-protective and anti-inflammatory effects after retinal IRI [14, 16, 17] or stroke [18, 19]. The application of CO-releasing molecules (CORM) represents a valuable alternative to CO inhalation because they can be administered in a streamlined way to biological systems, thereby significantly reducing toxic side effects to enhance safety. Pre- and postconditioning approaches with the molybdenum-based, water-soluble CORM ALF-186 have recently shown neuroprotective properties after ischemia [17, 20, 21]. Therefore, it is reasonable to explore the administration of CO directly into the vitreous, a common therapeutic route in ophthalmology.

While CO has been identified as a potent cell-protective molecule, the roles it plays in neuronal development and regeneration has been poorly understood. There is growing evidence that CO supports neurons in regenerating their axons. In their research, Scheiblich et al. were able to generate a gain in the neurite length of human model neurons using a CORM-2 to elevate exogenous CO levels [22]. However, blocking HO-1 activity did not cause any relevant changes to neurite elongation [22]. Estes et al. found a rapid and sustained increase in filopodial length in two neuron types from adult freshwater snails after exposure to CORM-2 [23], strengthening CO’s role as a modulator of growth cone motility and an effector of neurite pathfinding.

This in vivo study analyzed the effectiveness of intravitreally administered CORM ALF-186 after retinal IRI as a neuroprotective and neuroregenerative agent.

## Materials and methods

### Animals

Adult male and female Sprague-Dawley rats (1:1, 280-350g bodyweight, Charles River, Sulzfeld, Germany) were used in these experiments. Animals were fed with a standard diet *ad libitum*, being kept on a 12-h light/12-h dark cycle. All procedures involving the animals concurred with the statement of The Association for Research in Vision and Ophthalmology for the use of animals in ophthalmic and vision research and were approved a priori by the Committee of Animal Care of the University of Freiburg (Permit No: 35–9185.81/G-15/46 and G-16/79). All types of surgery and manipulations were performed under general anesthesia: 1. isoflurane/O_2_ for retrograde labeling with fluorogold (FG); 2. a mixture of intraperitoneally administered ketamine 50 mg/kg (Ceva-Sanofi, Duesseldorf, Germany) and xylazine 2 mg/kg (Ceva-Sanofi) for the ischemia-reperfusion-experiment. For topical corneal anesthesia one drop of proparacaine (Proparakain-POS 0.5%, Ursapharm, Saarbrücken, Germany) was given. To avoid exsiccation of the cornea, lubricant eye gel (Corneregel, Dexpanthenol 50mg/g, Bausch+Lomb, Berlin, Germany) was administered during and at the end of the procedure. Body temperature was maintained at 37 C±0.5°C with a heating pad. During recovery from anesthesia, the animals were placed in separate cages. The number of animals used for RGC quantification and molecular analysis was n = 6 per group, for retinal explants n = 7 per group. Animals were euthanized by carbon dioxide and eyes enucleated before analysis. Retinal tissue was harvested after IRI at t = 24h for analysis of mRNA- and protein-expression, at t = 2d or 7d for retinal explants, and t = 7d for RGC quantification. The right eyes remained unaffected during the experiment and were enucleated simultaneously with the left intervention eye.

### Retrograde labeling of RGC

Rats were anesthetized, placed in a stereotactic apparatus (Stoelting, Kiel, Germany). Retrograde RGC-labeling was done seven days before IRI. The scalp was cut open und retracted. The lambda and bregma sutures served as landmarks for drilling 3 holes on each site of the bregma sutures. A total amount of 7.8 μl fluorogold (FG) (Fluorochrome, Denver, CO, USA) dissolved in DMSO/PBS was injected into both superior colliculi through the drilling holes. To ensure adequate retrograde transport of FG into the RGC´s perikarya, further experimental interventions were done 7 days after retrograde labeling.

### Retinal ischemia-reperfusion-injury and treatment with intravitreal injection of ALF-186

Following randomization, rats were sedated and laid prone. Their heads were fixed and slightly bent towards the right to make the left eye easily accessible for IRI (iris in horizontal position). The anterior chamber of the left eye was cannulated with a 30-gauge needle connected to a reservoir containing 0.9% NaCl. Intraocular pressure was increased to 120 mm Hg for 60 minutes, and ocular ischemia was confirmed microscopically by interruption of retinal circulation. Reperfusion was initiated by removing the needle tip promptly. Rats without immediate recovery of retinal perfusion at the end of the ischemic period (n = 0) or those with lens injuries (n = 1) were excluded from the study since the latter prevents RGC death and promotes axonal regeneration [24]. To evaluate the neuroprotective effect of the CO released from ALF-186, animals received an intravitreal injection of 5 μl ALF-186 (dissolved in PBS, concentration: 5 mg/ml), PBS (vehicle control), or inactivated ALF-186 (iALF-186) using a Hamilton microliter syringe (10 μl) with a 30-gauge needle. After declining the needle to about 30°, intravitreous injections were performed under microscope visualization, using the inferior quadrant of the eye. Substances were injected immediately after IRI. Contralateral eyes remained unaffected and were used for comparison. ALF-186 ([Mo(CO)3(-histidinato)]Na, Alfama Inc., Lisbon, Portugal) was kindly provided by C. Romão [25] and was freshly dissolved in PBS prior to the treatment. The inactive compound (iALF) was prepared to exclude the crucial effects of the molybdenum heavy metal backbone on retinal cells by dissolving the ALF-186 in PBS and incubating it for 24 h under exposure to air and light. After 24 h, the solution was bubbled with nitrogen to remove residual CO.

### Western blot analysis of MAP-kinases

24 h after IRI retinal tissue was harvested for analysis of protein expression. Total protein from ¾ of retina was extracted and processed for Western Blot as described previously. The contralateral eyes were processed in the same manner. The membranes were blocked with 5% skim milk in Tween20/PBS and incubated in the recommended dilution of protein specific antibody (p-ERK1/2 #4370, p-p38 #9211, Cell Signaling Technology, Danvers, MA, USA) overnight at 4°C. After incubation with a horseradish peroxidase-conjugated anti-rabbit secondary antibody (GE Healthcare, Freiburg, Germany), proteins were visualized using the ECL Western blotting detection reagent (Western Lightning plus ECL, #NEL103001EA, PerkinElmer, Waltham, MA, USA) following the manufacturer´s instructions. Images were generated with Fusion Fx imaging system (PEQLAB Biotechnologie GmbH, Erlangen, Germany). For normalization, blots were re-probed with ERK1/2 and p38 (#4695, #9212, Cell Signaling Technology, Danvers, MA, USA). Blots were analyzed by laser scanning densitometry (Personal Densitometer; GE Healthcare).

### Real time polymerase chain reaction (RT-PCR)

From retinal tissue harvested 24 h after IRI, total RNA from ¼ of retina was extracted using a column-purification based kit (RNeasy Micro Kit, Qiagen, Hilden, Germany) according to the manufacturer´s instructions. Reverse transcription was performed with 50 ng of total RNA using random primers (High Capacity cDNA Reverse Transcription Kit, Applied Biosystems, Darmstadt, Germany). Real time polymerase chain reactions (RT-PCR) were done with TaqMan probe-based detection kit (TaqMan PCR universal mastermix, Applied Biosystems, Darmstadt, Germany). Following primers were used: Caspase-3 #Rn00563902_m1, NF-κB #Rn01399583_m1, Hsp-70 #Rn04224718_u1, Hsp-90 ##RN00822023_g1, AIF-1 #Rn00574125_g1, Gap-43 #01474579_m1, TNF-α #Rn01525859_g1 (all from Applied Biosystems, Darmstadt, Germany). The PCR assays were then performed on a RT-PCR System (StepOne, Applied Biosystems, Darmstadt, Germany) with the following cycling conditions: 95°C for 10 min, 40 cycles of 95°C for 10 sec and 60°C for 1 min. Reaction specificity was confirmed by running appropriate negative controls. Cycle threshold (CT) values for each gene of interest were normalized to the corresponding CT values for GAPDH (ΔCT). Relative gene expression in IRI injured retinal tissue either with ALF-186, PBS or iALF injection was calculated in relation to the corresponding gene expression in the contralateral non-injured retinal tissue of each individual animal (ΔΔCT).

### RGC quantification

Seven days after sinistral IRI, retinal tissue of both eyes was immediately harvested, placed in ice-cold Hank´s balanced salt solution and further processed for whole mount preparation. Retinae were carefully placed on a nitrocellulose membrane with the ganglion cell layer (GCL) on top. After removing the vitreous body, retinae were fixed in 4% paraformaldehyde for 1h and then embedded in mounting media (Vectashield; Axxora, Loerrach, Germany). The densities of FG-positive RGC were determined in blinded fashion using a fluorescence microscope (AxioImager; Carl Zeiss, Jena, Germany) and the appropriate bandpass emission filter (FG: excitation/emission, 331/418 nm). Briefly, we photographed 3 standard rectangular areas (0.200 mm x 0.200 mm = 0.04 mm2) at 1, 2 and 3 mm from the optic disc in the central regions of each retinal quadrant. Hence, we evaluated an area of 0.48 mm^2^ per retina. To calculate the average RGC density in cells/mm^2^, we multiplied the number of analyzed cells/0.04 mm^2^ with 25. Secondary fluorogold stained activated microglia cells (AMC) after RGC phagocytosis were identified by morphologic criteria and excluded from calculation. All data are presented as mean RGC densities [cells/mm^2^] ± SD.

### Retinal explants

The intrinsic ability of RGC to regrow axons after injuries has been previously demonstrated [26, 27]. This regenerative ability has been partially attributed to injury-induced upregulation of growth-associated proteins [28], molecules assembled to form growth cones and axons [29], and transcriptional activation [30] under the premise of a growth-permissive condition. In contrast, without a stimulating injury, adult RGC axons fail to regenerate in vitro following axotomy. To evaluate whether CO affects the intrinsic ability of RGC to regrow axons after IRI, retinal organ cultures were applied. The retinal tissue of intravitreally treated eyes (ALF or iALF) after IRI and the contralateral eyes were harvested after two or seven days and further processed for whole mount preparation (n = 7). Eight homogenous explants/retinae were trepanned (diameter 1.5 mm, mid-peripheral) and cultured in serum-free S4 media (Astrocyte Microglia Growth Medium, based on Needham et al. 1987 [31]) on a laminin-1-coated substrate for six days (RGC facing down), as previously described [27, 32, 33]. Half of the medium was subsequently changed every two days. The axons regrowing from the retinal explants were counted along the explant margin after six days in vitro following immunolabeling. Immunohistochemistry was used, following standard protocols to label the RGC-axons (anti-beta-III tubulin, polyclonal antibody, mouse, dilution 1:10.000, red, Promega, Madison, WI) growing out of the retinal explants and disclose the migrating Müller cells and astrocytes (anti-glial fibrillary acidic protein (GFAP), polyclonal antibody, rabbit, dilution 1:000, green; LabVision, Fremont, CA). Explants (n = 2 per group) were labeled with anti-GFAP and anti-nestin (anti-nestin, monoclonal antibody, mouse, 1:400; Millipore, Merck, Darmstadt, Germany). Nuclei of retinal cells were stained with 4’.6-diamino-2-phenylindole dihydrochloride hydrate (DAPI, Sigma, Taufkirchen, Germany) and added to the embedding medium (Mowiol; Calbiochem, San Diego, CA, USA).

Explants were examined circumferentially under a fluorescence microscope (Axio Vision Imager A1; Carl Zeiss, Jena, Germany), counting all beta-III tubulin positive axons which passed a defined distance of 200 μm to the explant margin. All photographs presented in this paper were taken with a confocal microscope (Leica TCS SP8 confocal microscope, Buffalo, IL, USA).

### Quantification of GFAP-positive glial cell migration and GFAP/beta-III tubulin coexpressing cells in culture

IRI activates glial cells and their reactivity, inducing both neuroprotective and neurodegenerative modes of action. Two types of macroglial cells can be found in the mammalian retina, astrocytes and Müller cells; they express GFAP as an intermediate filament protein. Although Müller cells express little or no GFAP in healthy rat retinae, they show increased GFAP expression after retinal injuries, including IRI [34]. To quantify GFAP-positive cell migration out of retinal explants seven days after IRI, photographs of the entire explants were taken with a fluorescence microscope (Observer Z.1; Carl Zeiss, Jena, Germany) using the stack and tile function and composed into mosaics. The total area of GFAP-positive cells which passed the explant margin was calculated into pixels with ImageJ using the threshold tool. The entire area of the trepanned explant was also evaluated into pixels to compensate for fluctuations in their size. The pixels of the GFAP-positive area were divided by the area of the explant to achieve the so-called GFAP-quotient. Results are expressed as the median GFAP-quotient in box plots for each treatment group seven days after IRI (n = 7).

Class III beta-tubulin (beta-III tubulin) is a microtubule element of the tubulin family, which is primarily expressed in neurons and might be involved in neurogenesis and axon guidance and maintenance. Normally, glial cells do not express beta-III tubulin, although the coexpression of this marker in fetal or reactive astrocytes has been reported [35, 36]. Double-immunolabeled cells (GFAP- and beta-III tubulin-positive cells) were detectable after culturing and counted circumferentially using ZEN-blue 2010 software. Results are expressed as median absolute numbers in box plots for each treatment group seven days after IRI (n = 7).

### Statistical analysis

Data of RGC-quantification and protein expression were analyzed as follows: A One-way-ANOVA with multiple comparison analysis (Tukey Contrasts) was used to compare the ischemic eyes and the intravitreal treatment. A paired t-test was used for comparisons between right and left eyes to take into account the dependent nature of these values. Data of mRNA were analyzed using a computerized statistical program (SigmaPlot Version 11.0, Systat Software Inc., San Jose, CA, USA). The results are presented as means (±SD) after normal distribution of the data had been verified. One-way ANOVA for repeated measurements was used for between-group comparisons with a post hoc Holm-Sidak test. P<0.05 was considered statistically significant. Axonal resprouting and glial migration out of the retinal explants were analyzed with the R-System. Results are presented in box plots as a median and quartiles. The Kruskal-Wallis-test and ANOVA with post hoc analysis were used to assess statistical significance. Again, P-values below 0.05 were considered statistically significant. The unoperated right eyes of experimental animals were used for comparison.

## Results

### Intravitreal ALF-186 injection protected retinal ganglion cells against ischemia-reperfusion-injury

**Fig 1:**
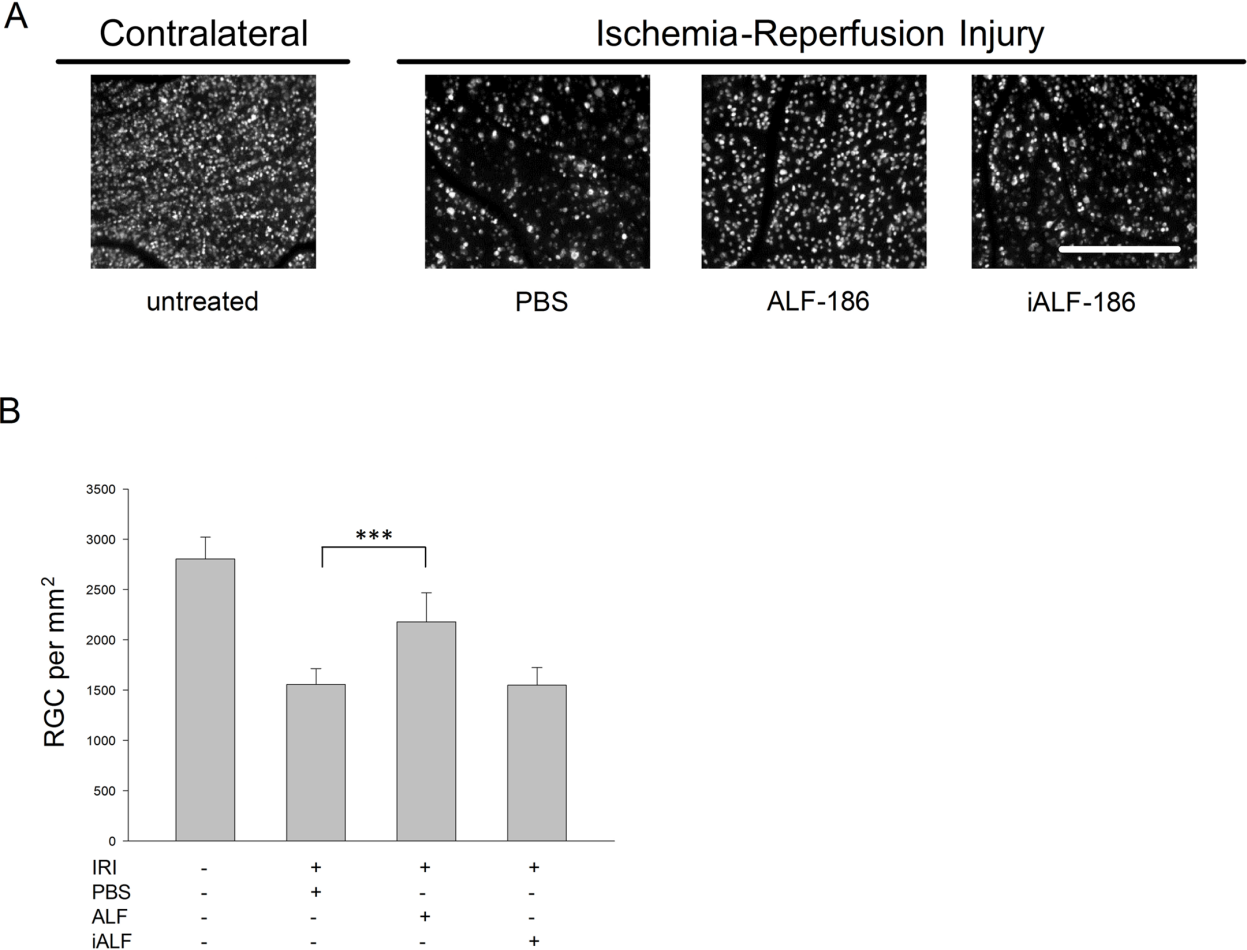
Protection of retinal ganglion cells (RGC) by intravitreal-administered ALF-186 after ischemia-reperfusion-injury (IRI). **(A)** Representative images of flat mounted retinae seven days after IRI to the left eye (left picture: untreated right eye without any treatment, picture 2–4: IRI eye after intravitreal injection of PBS, ALF-186, or iALF-186). RGC had been retrogradely labeled with fluorogold one week prior to intervention. A significant reduction of RGC-density was detectable after IRI and PBS or iALF treatment, while ALF protected RGC after IRI. Scale bar indicates 200 μm. **(B)** Quantification of RGC-density (cells/mm^2^; mean±SD; n = 6) revealed a marked protection of RGC after ischemia in the ALF-treated eyes in comparison to iALF or PBS. *** = p<0.001.

Compared to the RGC-density in the contralateral unoperated right eyes (2804±214 RGC/mm^2^; data are mean±SD; n = 6), IRI+PBS injection resulted in a remarkable loss of RGC (1554±159 RGC/mm^2^), p<0.001. Intravitreally injected ALF-186 immediately after IRI provided RGC protection and reduced the extent of RGC-damage in ischemic eyes (IRI+PBS 1554±159 vs. IRI+ALF 2179±286, p<0.001), Fig 1A and 1B. In contrast, administration of inactivated ALF-186 did not reduce RGC-death after ischemia (IRI+iALF 1596±307 vs. IRI+ALF 2179±286, p = 0.002). Thus, the effects of iALF and PBS were comparable (IRI+iALF-186 1596±307 vs. IRI+PBS 1554±159, a difference not statistically significant, Fig 1A and 1B).

### ALF-186 differentially regulated MAP kinase phosphorylation

**Fig 2:**
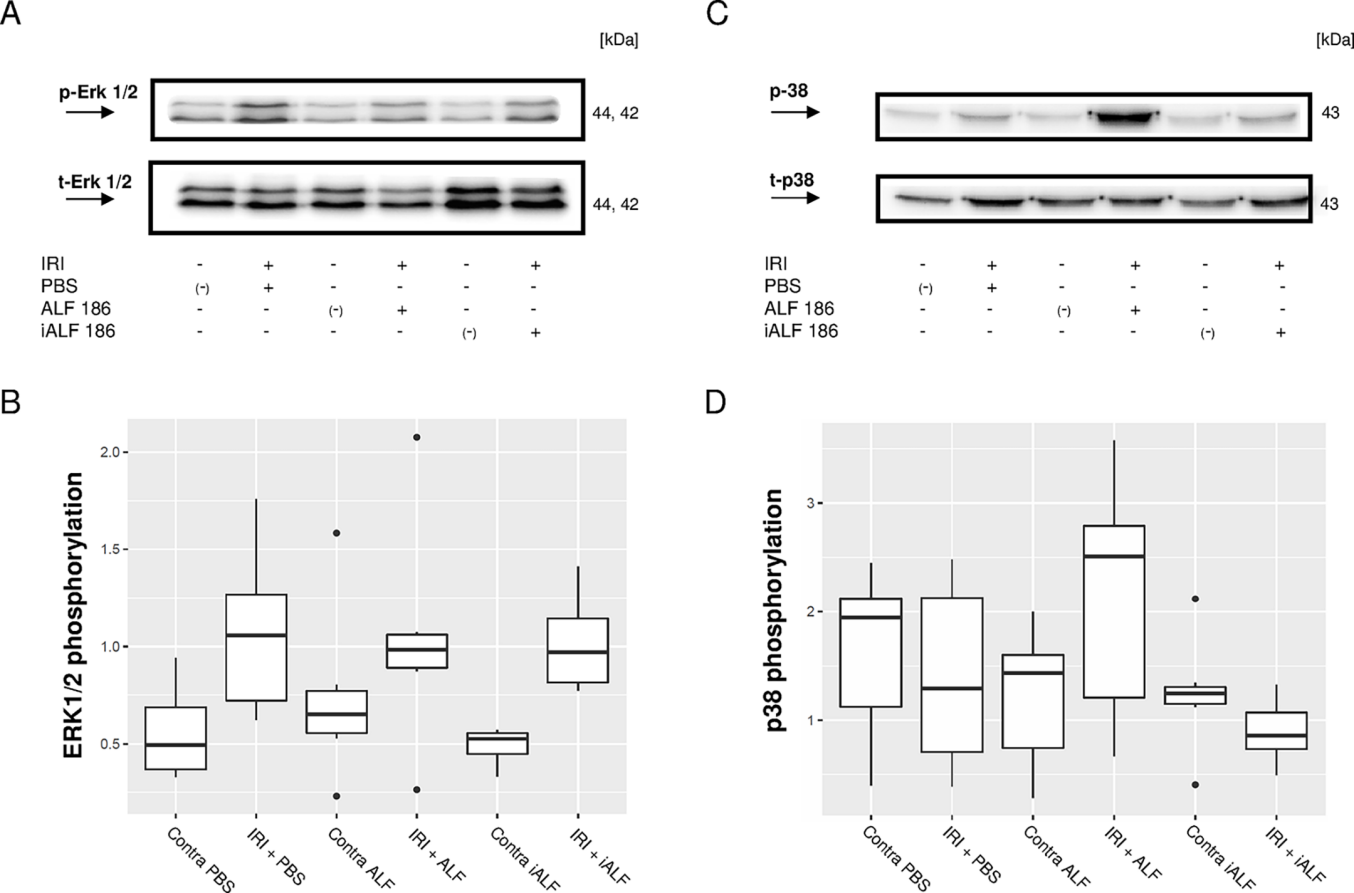
Effect of ALF-186 on mitogen-activated protein (MAP) kinases ERK1/2 and p38. **(A)** Representative western blot image showing the suppression of phosphorylated ERK1/2 compared to total ERK1/2 after IRI+ALF-186 treatment. The contralateral eye to each treated eye was indicated with (-) to demonstrate, that no MAP-kinase alterations were detectable in and between the right eyes. **(B)** Densitometric analysis of n = 6 western blots for phosphorylated ERK1/2 after IRI+treatment given in box plots. ALF-186 not affected the IRI-induced upregulation of ERK1/2, which was equally present after PBS, ALF or iALF-treatment, p>0.97. **(C)** Representative western blot image showing the increase of phosphorylated p38 compared to total p38 after intravitreal ALF-186 treatment. **(D)** Densitometric analysis of n = 7 western blots for phosphorylated p38 after IRI+treatment given in box plots. IRI+PBS vs. IRI+ALF-186, p = 0.267; IRI+ALF-186 vs. IRI+iALF-186, p = 0.032.

MAP kinases are an important component of intracellular signal transduction and are involved in apoptotic and survival processes. Thus, we analyzed the effect of intravitreal ALF-186 on MAP kinase phosphorylation in the retina of untreated eyes and IRI eyes after PBS, ALF, or iALF injection. The intravitreal injection of ALF-186 after ischemia not affected the IRI induced ERK1/2 phosphorylation (Densitometric analysis, n = 6 western blots, data are given in medians and shown in box plots; IRI+PBS 1.057 vs. IRI+ALF-186 0.984, p = 0.992, Fig 2A and 2B). iALF-186 had no independent effect on ERK1/2 phosphorylation, which did not differ significantly from the PBS-treated group (IRI+iALF-186 0.971 vs. IRI+ALF-186 0.984, p = 0.972, Fig 2A and 2B). In contrast to ERK1/2, we detected an increase in p38 phosphorylation after ALF-186 treatment, while treatment with iALF and PBS showed no alteration (IRI+PBS 1.29 vs. IRI+ALF-186 2.51, p = 0.267; IRI+ALF-186 2.51 vs. IRI+iALF-186 0.86, p = 0.032, Fig 2C and 2D).

### ALF-186 inhibited IRI-induced Caspase-3 and NF-kappaB mRNA expression

**Fig 3:**
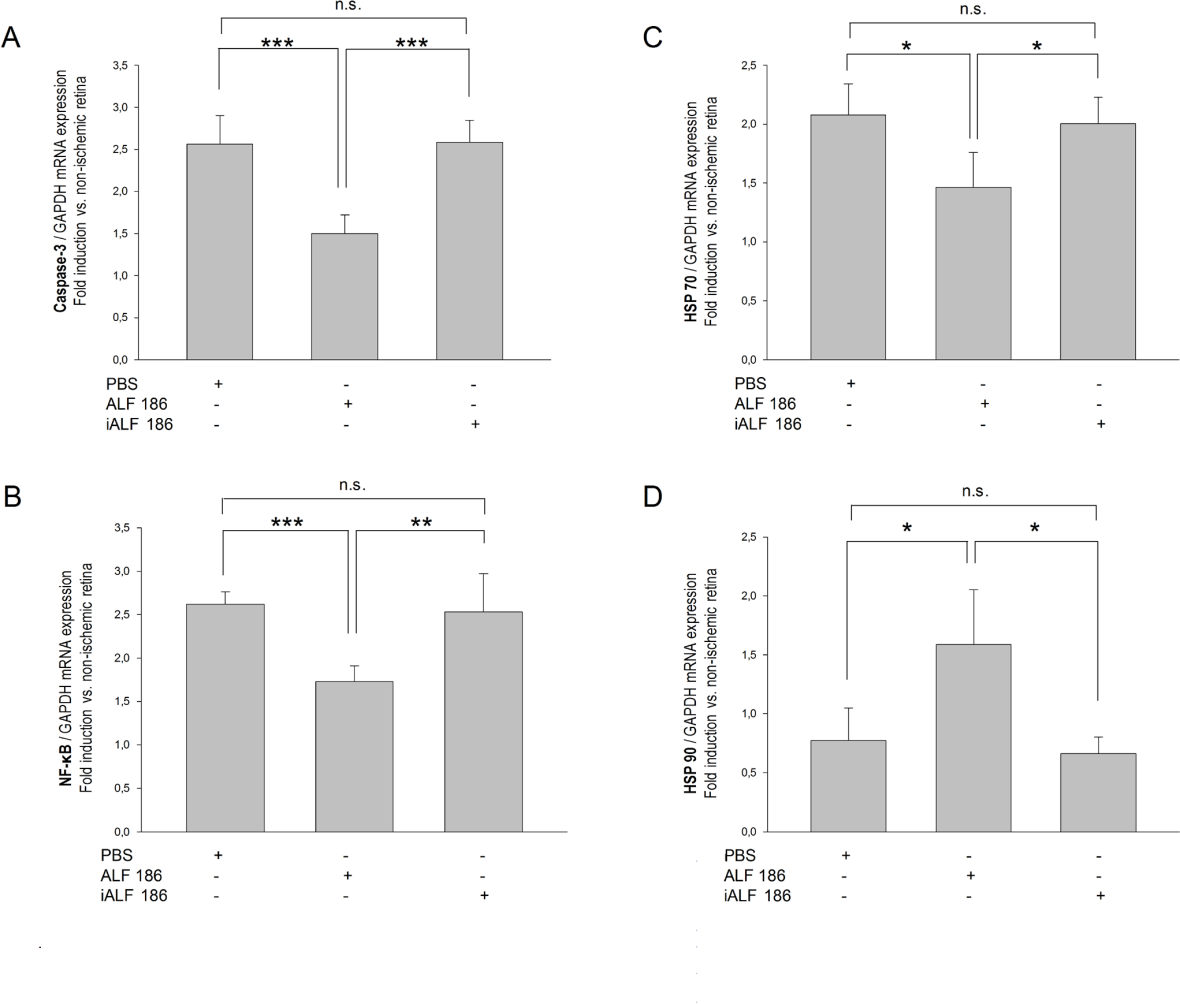
Intravitreal ALF-186 decreased the IRI-induced capsase-3 and NF-κB mRNA expression and affected the heat-shock response differently. **(A)** Fold induction of caspase-3 mRNA expression after PBS, ALF-186, or iALF injection after IRI compared to GAPDH in relation to the corresponding non-ischemic contralateral retina analyzed by RT-PCR (n = 6; mean±SD; *** = p<0.001; *** = p<0.001). ALF decreased the IRI-induced caspase-3 expression significantly. **(B)** Fold induction of NF-κB mRNA expression after PBS, ALF-186, or iALF injection+IRI (n = 6; mean±SD; *** = p<0.001; ** = p<0.01). Intravitreal CO suppressed NF-κB mRNA expression after IRI. **(C+D)** Fold induction of HSP-70 (C) and HSP-90 (D) mRNA expression 24 h after IRI (n = 6; mean±SD; * = p<0.05). While HSP-70 expression was decreased, HSP-90 was induced by ALF.

Next, we performed RT-PCR, analyzing caspase-3 mRNA expression. ALF-186 injection decreased IRI-induced caspase-3 mRNA expression significantly (n = 6; data are mean±SD; IRI+PBS 2.56±0.34 vs. IRI+ALF-186 1.50±0.22, p<0.001, Fig 3A). Inactivated ALF after IRI neither increased nor decreased caspase-3 induction (IRI+ALF-186 1.50±0.22 vs. IRI+iALF-186 2.58±0.26, p<0.001). Similarly, transcription factor NF-kappaB, which plays an important role in processes involved in inflammation and cell death, decreased with the ALF-186 injection (IRI+PBS 2.62±0.14 vs. IRI+ALF-186 1.73±0.18, p<0.001). iALF did not alter NF-kappaB (IRI+ALF-186 1.73±0.18 vs. IRI+iALF-186 2.53±0.44, p<0.01, Fig 3B).

### ALF-186 affected the heat shock proteins HSP-70 and HSP-90 differently

We examined the effect of ALF-186 on the heat shock responses HSP-70 and HSP-90. Opposing effects were observed: injection of ALF-186 immediately after reperfusion reduced HSP-70 mRNA expression (n = 6; data are mean±SD; IRI+PBS 2.08±0.26 vs. IRI+ALF-186 1.47±0.29; IRI+ALF-186 1.47±0.29 vs. IRI+iALF-186 2.01±0.22, both p<0.05, Fig 3C) but increased HSP-90 expression (IRI+PBS 0.77±0.28 vs. IRI+ALF-186 1.59±0.46; IRI+ALF-186 1.59±0.46 vs. IRI+iALF-186 0.66±0.14, both p<0.05, Fig 3D).

### ALF-186 mitigated IRI-induced AIF-1 and TNF-α mRNA expression and increased Gap-43

**Fig 4:**
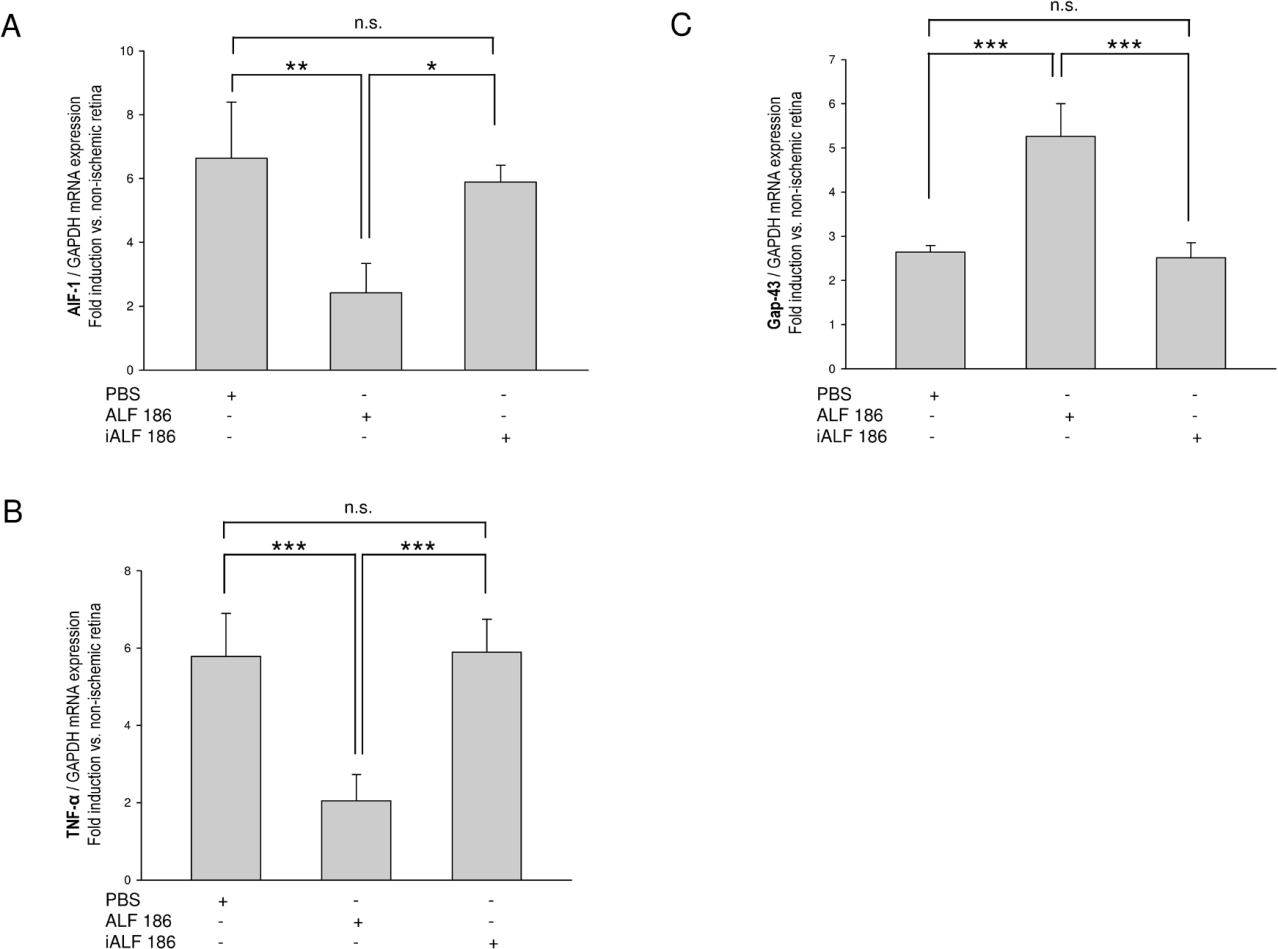
Effect of ALF-186 on AIF-1, TNF-α, and Gap-43 mRNA expression. **(A)** Fold induction of AIF-1 mRNA expression after IRI (n = 6; data are mean±SD; ** = p<0.05; * = p<0.05). **(B)** Fold induction of TNF-α mRNA expression after ALF-186 (n = 6; data are mean±SD; *** = p<0.001). **(C)** Fold induction of Gap-43 mRNA expression after ALF-186 (n = 6; data are mean±SD; both *** = p<0.001).

The allograft inflammatory factor-1 (AIF-1), also known as ionized calcium-binding adapter molecule 1 (Iba1), is a protein that is associated with microglial activation. We observed an AIF-1 downregulation by ALF-186 (n = 6; data are mean±SD; IRI+PBS 6.64±1.76 vs. IRI+ALF-186 2.42±0.92, p<0.05; IRI+ALF-186 2.42±0.92 vs. IRI+iALF-186 5.89±0.53, p<0.05, Fig 4A). Furthermore, the inflammatory cytokine TNF-α was suppressed by ALF (IRI+PBS 5.78±1.12 vs. IRI+ALF-186 2.05±0.68; IRI+ALF-186 2.05±0.68 vs. IRI+iALF-186 5.89±0.85, both p<0.001, Fig 4B).

Gap-43 is associated with nerve growth during development and regeneration in the CNS and optic nerve and is a major component of the motile growth cone that forms the tips of elongating axons. Gap-43 synthesis decreased with maturation [37] but could be re-expressed in RGC after injury [5, 38]. Analysis of Gap-43 mRNA showed an increased expression in association with ALF-186 treatment after retinal IRI (IRI+PBS 2.64±0.15 vs. IRI+ALF-186 5.26±0.74; IRI+ALF-186 5.26±0.74 vs. IRI+iALF-186 2.51±0.34, both p<0.001, Fig 4C).

### ALF-186 augmented the IRI-induced regenerative response of RGC axons in vitro

**Fig 5:**
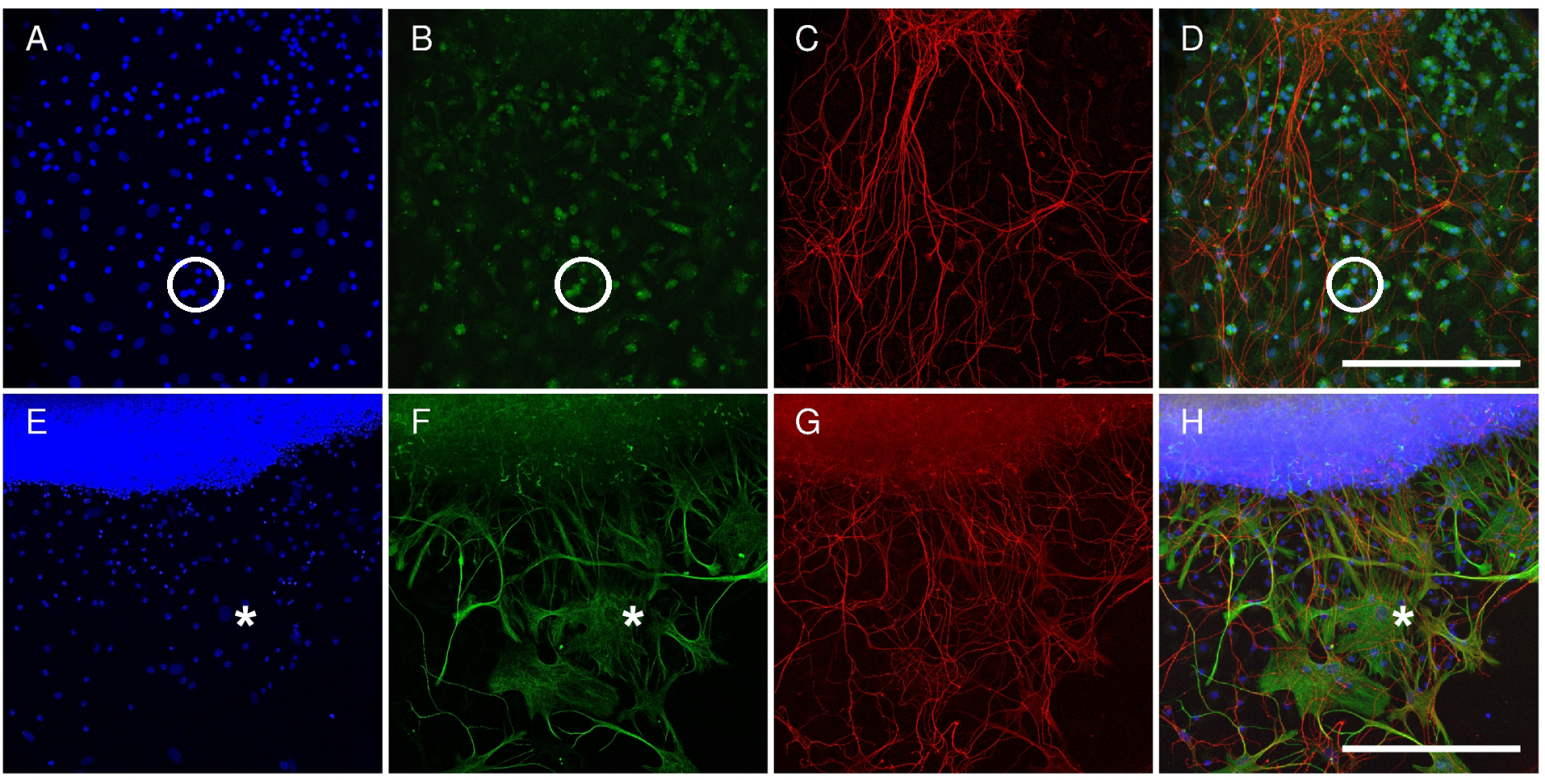
Immunohistochemical analysis of cells migrating out of ischemic retinal explants. Retinal explants were transferred onto laminine-1 coated cultures for six days to allow RGC regeneration. Next, explants were analyzed by immunohistochemistry. Different cell types migrated out of the retinal explants during the time in culture. **(A+E)** Two types of DAPI-positive nuclei were apparent: smaller (circle) and bigger (asterisk). **(B+D)** The smaller nuclei belonged to iba-1 positive microglia cells (circle). **(F+H)** The bigger nuclei belonged to GFAP-positive Müller cells and astrocytes (asterisk). **(C+G)** Both glial cell types did not compromise the growth behavior of the beta-III tubulin positive RGC axons. Scale bar upper row 300 μm, lower row 600 μm.

Lens injury, optic nerve crush and glaucoma induced a regenerative response in RGC-axons when transferred into a growth-permissive environment [24, 32, 33]. Here we investigated the IRI-induced regenerative response of RGC-axons in vitro and if intravitreal CO influences their regenerative capacities. At day six in vitro, the explants were analyzed after immunohistochemistry. Different cell types migrated out of the ischemic retinal explants during the time in culture (Fig 5); there were two types of DAPI-positive nuclei: smaller and bigger (Fig 5A and 5E). The smaller nuclei belong to iba-1-positive microglia cells (circle in Fig 5B and 5D). The bigger nuclei belong to GFAP-positive Müller cells and astrocytes (asterisk in Fig 5F and 5H). Both glial cell types did not compromise the growth behavior of the beta-III tubulin-positive RGC axons (Fig 5C and 5G). Rather, it looked as if the RGC axons and GFAP-processes used each other as a guidance system, showing high affinity.

**Fig 6:**
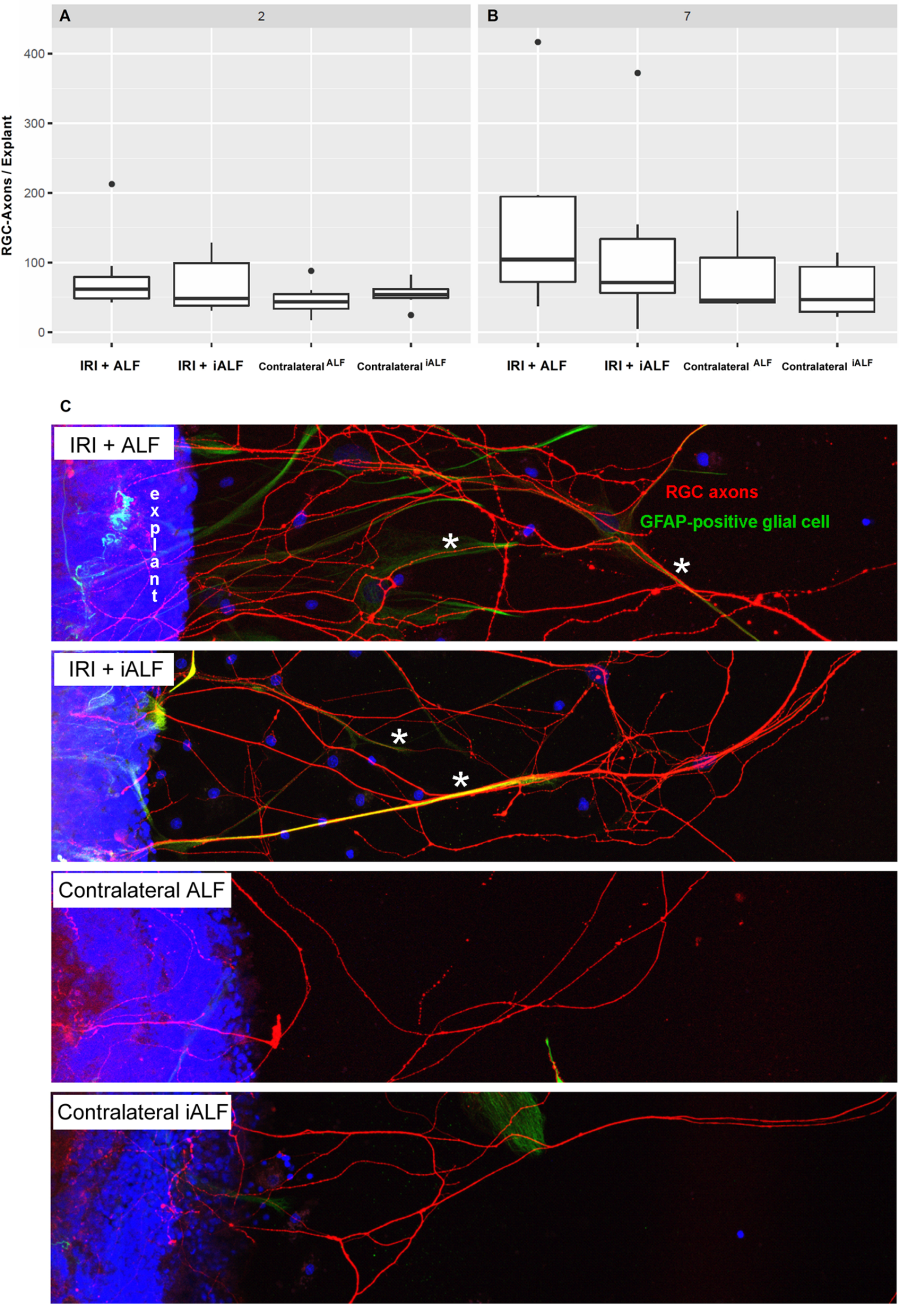
ALF-186 augmented the IRI-induced regenerative response of RGC-axons in vitro. **(A+B)** Box-Whisker-Plots showing the amount of regenerated RGC axons per explant for all treatment groups (n = 7 retinae, averaged value of 5–8 adhered explants per retina) explanted two (A) or seven (B) days after IRI. The growth of RGC axons was more pronounced in the ischemic eyes compared to contralateral eyes and stronger after seven than two days post IRI. Furthermore, axonal resprouting was tendentiously strongest after IRI and ALF-injection, without reaching statistical significance compared to IRI+iALF. **(C)** Representative pictures of regenerated RGC axons of all groups seven days after IRI. Quantification was performed using fluorescence microscopy (Zeiss ApoTome) to count the beta-III tubulin positive axons (red) after six days in culture which passed a defined distance of 200 μm to the explant margin. Cell nuclei were stained with DAPI (blue). GFAP-positive processes (green) were discriminated from RGC axons by immunolabeling. The RGC axons and GFAP-processes used each other as a guidance system, showing high affinity (asterisks).

The amount of regenerated axons was low two days after intervention and did not differ significantly between groups (Fig 6A) (median axons per explant of n = 7 retinae: IRI+ALF 62.00, IRI+iALF 48.25, contralateral^ALF^ 43.40, contralateral^iALF^ 53.66, p>0.05). The regenerative response increased in ischemic eyes in vivo seven days after IRI (Fig 6B and 6C, median axons per explant of n = 7 retinae: IRI+ALF 104.25, IRI+iALF 71.29, p = 0.92). Thus, the regenerative propensity was greatest in IRI+ALF treated explants. In the contralateral unoperated eyes, the regenerative response remained low, representing the effect of axotomy during the explant procedure (Fig 6B, median axons per explant of n = 7 retinae: contralateral^ALF^ 46.00 vs. contralateral^iALF^ 46.94, p = 0.99). The regenerative response and the RGC-density were inversely related seven days after IRI in the contralateral retinae and after IRI+iALF (high RGC densities and low regeneration, see Fig 1). Although significantly less RGC-damage was induced in the IRI+ALF group, this group regenerated most powerful (Fig 6C upper row).

### ALF-186 influenced the extent of GFAP-positive glial cell migration out of retinal explants

**Fig 7:**
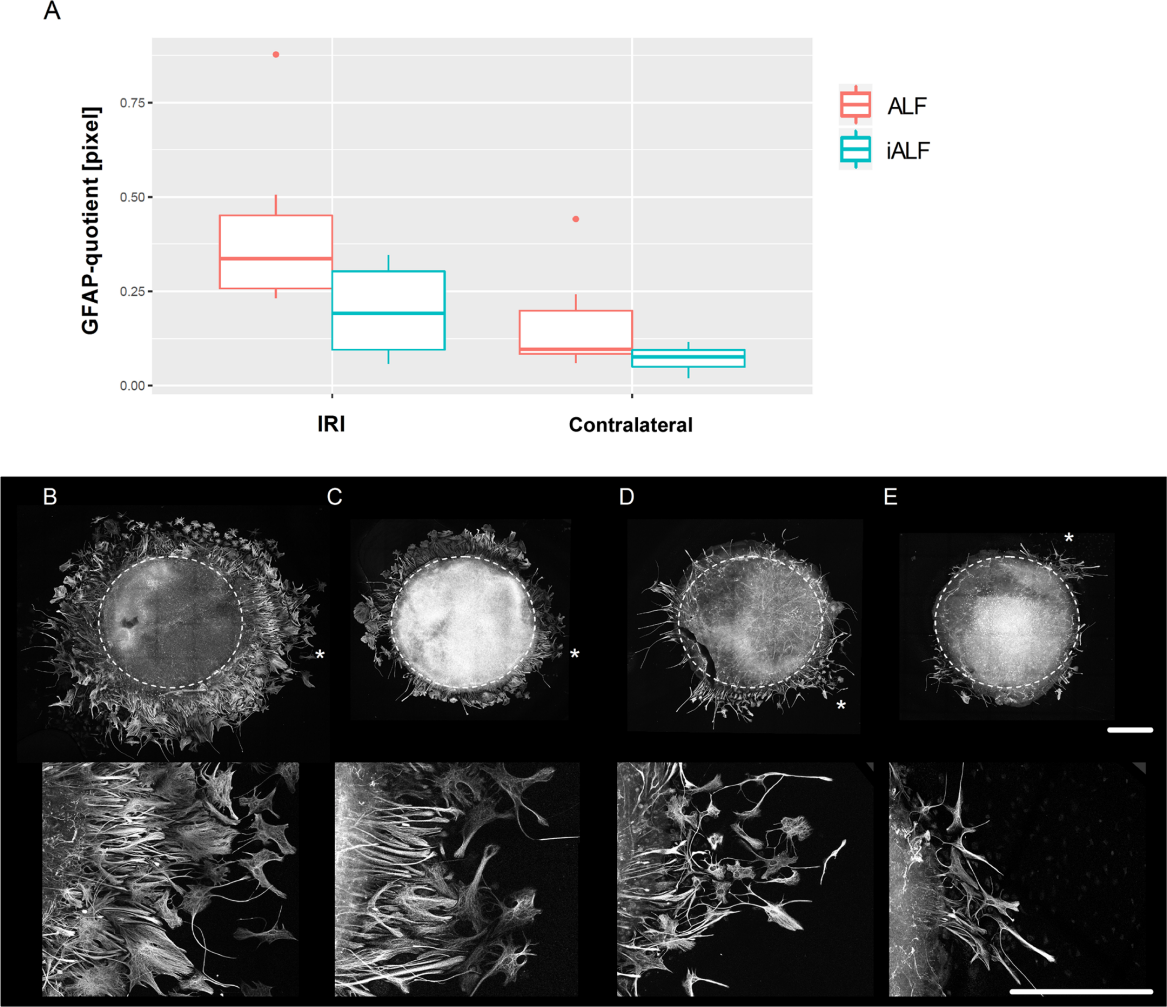
ALF-186 induced the extent of GFAP-positive glial cell migration out of the retinal explants. **(A)** The amount of GFAP-positive migrating glial cells was highest in the IRI+ALF treated group, followed by the IRI+iALF treated group at day six in vitro (p = 0.06). A slight GFAP-reactivity could also be found in the contralateral retinae after ALF-injection into the left eye (contralateral^ALF^) compared to contralateral^iALF^ (p = 0.15). **(B-E)** Representative explants in lower and higher magnification of all treatment groups on day seven post IRI. The massive migration of GFAP-positive cells was obvious in the IRI+ALF group (B) and descended from IRI+iALF (C) to contralateral^ALF^ (D) to contralateral^iALF^ (E). The asterisk beside the explant-mosaic marked the origin of the pictures in the lower row, showing this location in higher magnification. Two types of GFAP-positive cells were distinguishable beside the explant margin: 1) longitudinal-shaped cells remaining firmly anchored to the explant, frequently without a visible cell nucleus but a dense filamentous appearance; 2) star-shaped cells with an expansive cytoplasm and prominent centralized nucleus, unconnected and farther from the explant with several processes. Scale bars in both rows 600 μm. Upper row: original tile 581.25 x 581.25, identifiable in the mosaic-reconstruction of the explants; explant-diameter trepanned was 1.5 mm, the circumference of the explant was marked (white dashed line).

We recognized a remarkable macroglial migration out of retinal explants after IRI (Fig 7). Therefore, we investigated the area of GFAP-positive cells surrounding the retinal explants seven days after IRI and ALF or iALF-injection. At day six in vitro, the area of GFAP-positive glial cell migration was more expansive in the IRI+ALF-treated retinae than the IRI+iALF-treated retinae (median GFAP-quotient 0.34 vs. 0.19, respectively, n = 7, p = 0.06), Fig 7A–7C. Interestingly, a minor tendency of increased GFAP-positive glial cell migration was seen in the contralateral retinae after ALF-injection into the left eye (contralateral^ALF^) compared to contralateral^iALF^ (GFAP-quotient 0.10 vs. 0.08, respectively, n = 7, p = 0.15), Fig 7A, 7D and 7E). Morphologically, two types of GFAP-positive cells were found beside the explant margin: 1) cells with a longitudinal shape, remaining firmly anchored to the explant and frequently without a visible cell nucleus but a dense filamentous appearance; 2) star-shaped cells with several processes, with an expansive cytoplasm and prominent centralized nucleus, unconnected and in greater distance to the explant margin.

### GFAP and beta-III tubulin coexpressing cells in culture

**Fig 8:**
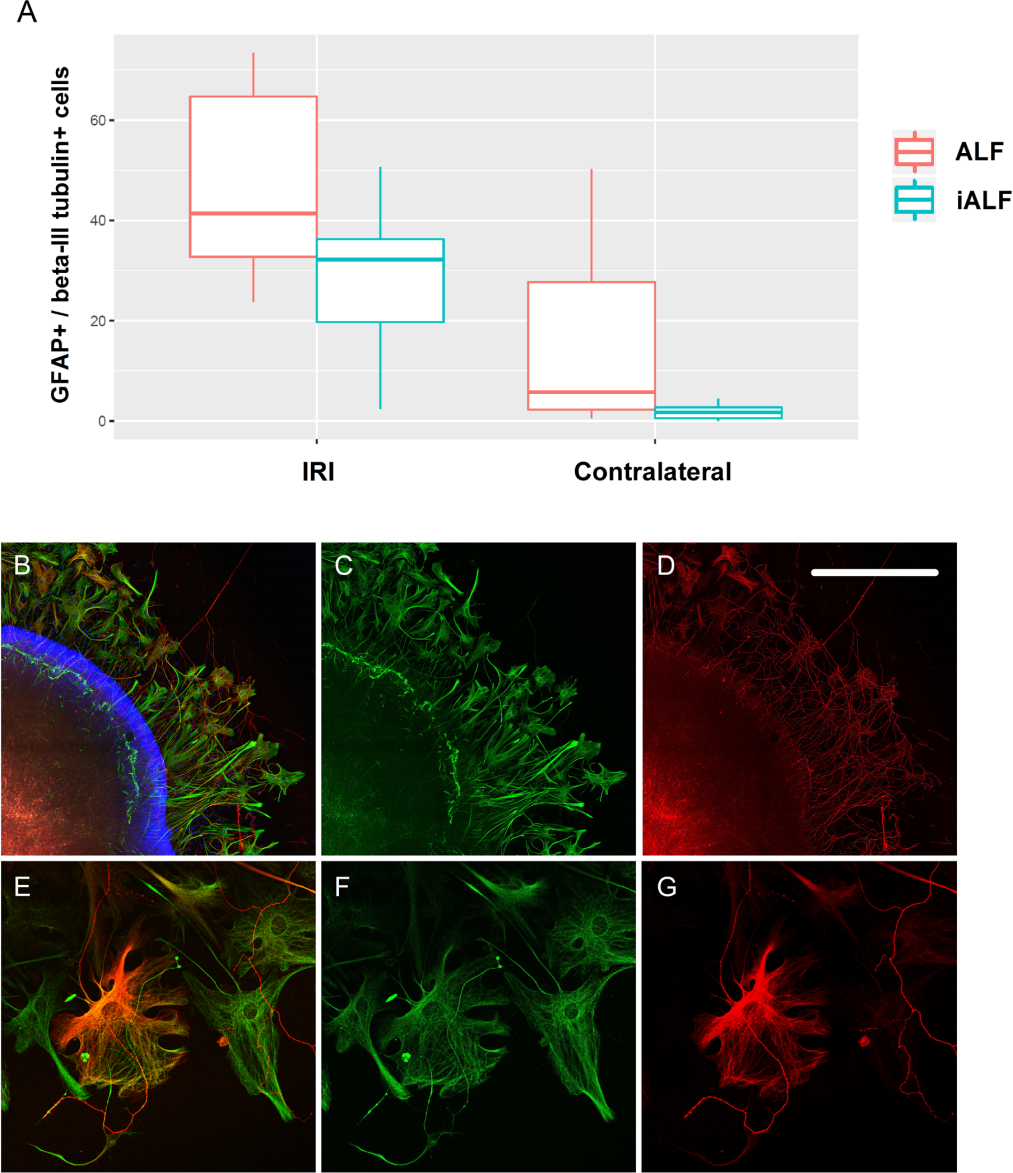
ALF-and IRI-induced beta-III tubulin immunoreactivity in GFAP-positive cells in culture. **(A)** Box-Whisker-Plots showing the amount of GFAP and beta-III tubulin coexpressing macroglial cells that migrated out of the retinal explants. The median amount of GFAP+ and beta-III tubulin+ cells per explant was significantly higher in both IRI groups and can be particularly found in the IRI+ALF group. **(B-D**) Retinal explant after IRI+ALF. GFAP (green, C) was expressed in all the migrating Müller cells and astrocytes. Some of these cells and the RGC-axons were beta-III tubulin immunopositive (red, D). **(E-G)** Immunoreactivity was located in different cytoskeletal areas. While GFAP-positive structures showed filamentous patterns and were concentrated perinuclearly and at the margin of cells (F), the beta-III tubulin was associated with microtubules and predominantly found in the perinuclear region (G). Scale bars B-D 600μm.

Coincidentally, we detected beta-III tubulin coexpression in some GFAP-positive macroglial cells that emerged from the retinal explants (Fig 8A and 8B–8D). We therefore wanted to know if IRI and ALF-186 affected the quantity of these cells. We found that the median amount of GFAP+ and beta-III tubulin+ cells per explant was significantly higher in both IRI groups compared to the contralateral eyes (IRI+ALF 41.43 vs. contralateral^ALF^ 5.75, n = 7, p = 0.03; IRI+iALF 32.25 vs. contralateral^iALF^ 1.71, n = 7, p = 0.002) at day six in vitro. While coexpressing cells were predominantly found in the explants of the ALF group compared to the iALF group, the differences were not statistically significant (IRI+ALF vs. IRI+iALF, p = 0.129; contralateral^ALF^ vs. contralateral^iALF^, p = 0.06, Fig 8A). The GFAP and beta-III tubulin immunolabeling were located in different cytoskeletal areas (Fig 8E–8G). Whereas GFAP-positive structures showed filamentous patterns and were concentrated perinuclearly and at the margin of all the migrating macroglia cells (Fig 8F), beta-III tubulin was associated with microtubules and was predominantly found in the perinuclear region of only isolated cells (Fig 8G).

To screen for additional hints of transdifferentiation in retinal glial cells migrating out of the retinal explants, we performed immunohistochemistry with anti-nestin and again found a partial match with GFAP (S1 Fig).

## Discussion

This studies main findings are as follows: (1) Intravitreal treatment with the CORM ALF-186 reduced the loss of RGC after IRI, reflecting its neuroprotective properties. (2) Intravitreal ALF-186 mediated anti-apoptotic signaling via p38 and caspase-3 but not ERK1/2. (3) Intravitreal ALF-186 showed anti-inflammatory capacities by reducing NF-kappaB, TNF-α, and AIF-1 mRNA expression after IRI. (4) ALF increased the re-expression of Gap-43 in ischemic retinae and provided further impetus to RGC’s regenerative response in culture. (5) ALF increased the IRI-induced reactivity and migration of GFAP-expressing retinal glial cells out of the retinal explants and to some extent their transdifferentiation, which was detected by the re-expression of neuronal markers.

The therapeutic potential of the HO-1/CO pathway in neurological diseases has been shown after stroke and in patients with multiple sclerosis and Alzheimer’s [9, 18, 19, 39]. CO is therefore a candidate for future therapeutic benefits [40, 41]. In the first part of this study, we demonstrated that even topical CORM injection into the vitreous body leads to sustained neuroprotective effects in the context of retinal IRI, which has not yet been shown. As recently detected with intravenous application of ALF-186 after IRI [17, 21], intravitreal ALF-186 spreads its effects on ischemic RGC in a comparable way and effective power. Intravitreal injection of ALF-186 immediately after reperfusion reduced the extent of RGC loss, corresponding to higher RGC density. This data correspond to the decreased caspase-3 expression in retinae after IRI+ALF compared to IRI+iALF or IRI+PBS, representing the anti-apoptotic capacity of ALF. As the mRNA results correspond excellent between intravitreal and intravenous administration [17, 21], protein cleavage of Caspase-3 was not further analyzed in this study. CO’s ability to protect RGC from injury by inhibiting caspase-3-dependent apoptosis was recently reported by Chen et al. [42] and in previous studies from our group [13, 14, 17, 21].

Intracellular signaling after IRI+ALF intravitreally likewise involved the gene suppression of NF-κB and TNF-α as well as the induction of p38 and HSP 90, as previously described and discussed in detail after intravenous ALF application [17, 21]. In both studies, corresponding changes on the level of protein were displayed; we thus decided not to confirm our mRNA results reported here by western blots, although the later would be methodically more valuable. Consistent with our results, Zheng and Zuo reported a p38 MAPK activator to induce neuroprotection and a p38 MAPK inhibitor to block an isoflurane-preconditioning-induced protection after transient middle cerebral arterial occlusion [43]. Similar results from an in vitro study showed that early activation of p38 MAPK was necessary to protect cultured L929-cyt16 cells from TNF-α-induced cytotoxicity [44]. The downregulation of AIF (Iba-1), a protein associated with microglial activation, is consistent with these findings, highlighting ALFs anti-inflammatory properties. While the amount of microglia activation was not quantified in this study, CO’s capacity to decrease microglia reactivity after IRI has been shown previously [16]. Further investigation on the role of microglial activation should be pursued.

Since we did not find any difference between the effects of PBS or iALF, it can be reasonably assumed that protection is mediated by CO alone and not by the CO-binding molybdenum carrier itself. Therefore, we only used iALF in control experiments later in our study.

In the second part of this manuscript, ALFs potential as an inductor of the RGC`s intrinsic regenerative potential after IRI was analyzed. Adult mammalian RGC fail to regenerate their axons after axotomy in vivo and in vitro [6]. They lose their intrinsic regenerative potential during postnatal maturation of the visual pathway [26]. Furthermore, the composition of glial cells in the environment of the RGC has been shown to reduce their capability to regenerate by the formation of glial scar tissue after injury (reviewed in [45]). Nevertheless, injuries to the axon or cell body itself initiate a series of changes in RGC crucial for neuroregeneration. These changes include an increase in RNA and protein synthesis, accompanied by the re-expression of growth-associated proteins (GAP) [46]. Acute optic nerve damage and glaucoma had been proven to induce a regenerative response in RGC-axons in a growth-permissive environment [24, 32, 33]. This study analyzed if IRI also stimulated RGC to regenerate their axons in a retinal organ culture system and if this response is modified by ALF treatment. As expected, the growth of RGC axons was more pronounced in the ischemic eyes than the contralateral eyes (Fig 6). Furthermore, this regenerative potential was stronger seven days post IRI than two days post IRI, which may reflect that important regeneration-associated mechanisms proceed in RGC in the days after ischemia. Interestingly, axonal resprouting was strongest after IRI and ALF-injection in comparison to IRI+iALF, we thus concluded that ALF in this concept of retinal culture worked as an additional inductor of the RGC`s intrinsic regenerative potential. The number of regrowing RGC axons could not be sufficiently explained by the number of surviving RGC, which significantly varies between groups 7 days after IRI. In the contralateral eyes (axotomized by the explant procedure) with high RGC content (2804 RGC/mm^2^) the regenerative capacity was lowest, in the IRI+iALF group with lowest RGC density (1596 cells/mm^2^) the regenerative response was high and after IRI+ALF (2179 RGC/mm^2^) even higher. Thus, the IRI procedure transferred the surviving but in number reduced RGC into a regenerative state in culture. ALF itself contributes a regenerative stimulus in addition to IRI, which is perhaps in accordance with the increased Gap-43 mRNA expression by ALF (Fig 4C), which needs further functional investigation. The fact that CO supported neurons to regenerate their axons has been previously reported by others. Scheiblich et al. were able to generate a gain in the neurite length of human model neurons using a CORM-2 [22]. Estes et al. found a rapid and sustained increase in filopodial length in two neuron types from the adult freshwater snail after exposure to CORM-2 [23], strengthening CO’s role as a modulator of growth cone motility and effector of neurite pathfinding. Enhanced expression of HO-1 in the dorsal root ganglion (DRG) cells of rats fostered neurite outgrowth after treatment with an iron chelator [47]. CO’s role in stimulating RGC to regenerate their axons after injury needs to be characterized in detail in further investigations.

Two types of macroglial cells exist in the mammalian retina, astrocytes and Müller cells. Müller cells (specialized radial glial cells) are the principal glial cell of the retina and extend from the inner limiting membrane to the outer limiting membrane. Both Müller cells and retinal neurons arise from a single progenitor cell of the neuroepithelium—although in two consecutive phases, with Müller cells being produced in the second phase after the RGC [48]. The developing neurons and Müller cells must migrate inward to their final retinal position, and the Müller cell processes and trunks guide many of the neurons and direct their differentiation. Müller cells provide functional and structural support to the retinal neurons (e.g., potassium siphoning, homeostasis control, release of neuroactive substances, and glutamate metabolism [49]). Astrocytes enter the developing retina from the brain along the developing optic nerve and subsequently migrate to the nerve fiber and ganglion cell layers [50]. They play a pivotal role in neuronal signaling and maintaining endothelial barrier properties [51]. Müller cells and astrocytes are remarkably resilient to damage and respond to retinal injury and diseases by changing their morphology, biochemistry, and physiology [45, 52]. This injury response, often referred to as reactive gliosis, can be beneficial to neurons by preventing glutamate neurotoxicity and releasing a variety of factors that protect neurons from cell death (e.g., neurotrophic factors [7, 53], growth factors, cytokines, erythropoietin [54], and antioxidant agents like HO [55]).

GFAP is a 51-kDa intermediate filament protein found in both retinal astrocytes and Müller cell end-feet and processes. Although Müller glial cells in normal rat retinae express little or no GFAP [56], they showed increased GFAP expression in retinal injuries including ischemia [13, 14, 57] and glaucoma [58, 59]; this expression occurs within the first 24 hours [60]. This study found remarkable reactivity and migration of GFAP-expressing cells out of retinal explants after IRI (Fig 7). Such an enormous amount of migrating GFAP-positive cells was not experienced when applying retinal explants after optic nerve crush or elevated intraocular pressure [32, 33]. This observation may correlate with an extended ischemic injury to all the inner retinal layers by the IRI-model used in this study compared to optic nerve injury models. The induced expression of GFAP in migrating Müller cells and astrocytes possibly suggests a potential neuroprotective mechanism in response to IRI, as we observed high affinity of these cells with beta-III tubulin-positive RGC-axons. Furthermore, the IRI-induced glial and regenerative response appeared both potentiated in the ALF treated group, where anti-apoptotic and anti-inflammatory mechanisms simultaneously proceed (Fig 3A and 3B, Fig 4A and 4B), and more RGC survived. Recent studies have found a promising growth-promoting role of retinal Müller cells and astrocytes, which were found to stimulate RGC-regeneration in vitro [7, 61]. Müller et al. reported that lens injury strongly induced the expression and release of ciliary neurotrophic factor (CNTF) from retinal astrocytes, which activated the JAK/STAT3 pathway in regenerating RGC [7]. Furthermore, transplantation of retinal glia enhanced the regeneration of DRG axons after root crush in adult rats [62]. Two recently released studies discovered that CO prevented neuronal cell death by targeting astrocytic metabolism through purinergic signaling and Ca2+-mediated PGC-1α/ERRα activation [63, 64]. The exact mechanism by which the reactive GFAP-positive cells in our culture model promoted RGC to regenerate their axon remains to be elucidated.

During the examination of axonal regrowth by counting beta-III tubulin positive RGC axons in our cultures, we unexpectedly encountered some beta-III tubulin-expressing Müller cells and astrocytes. The amount of GFAP and beta-III tubulin coexpressing macroglial cells migrating out of the retinal explants was significantly higher in both IRI groups and was particularly pronounced in the IRI+ALF group (Fig 8). Furthermore, contralateral eyes to ALF+IRI showed tendentiously more GFAP+/tubulin+ cells. It is therefore tempting to speculate that this beta-III tubulin expression in proliferating Müller cells and astrocytes was a sign of transdifferentiation. Unlike neurons, Müller cells have a life-long capability to dedifferentiate and reenter the proliferation cycle. Müller cells in culture display a distinct transdifferentiation into neuron-like cells, which is also observed under pathological conditions in vivo [65]. Previous studies reported the expression of neuronal or ganglion cell-specific markers in Müller cells after injury (e.g., Thy-1 [66], neurotransmitter phenotypes [67, 68], and Brn3 [69]). Beta-III tubulin, widely regarded as a neuron-specific marker, was recently found constitutively expressed in fetal astrocytes of the human brain [35]. Similarly, beta-III tubulin expression has also been reported in Müller glia cell lines, where retinal Müller glia have been assigned the role of multipotent neural stem cells (NSC) [70]. An alternative interpretation is that the coexpression of beta-III tubulin in GFAP+ retinal glial cells may reflect developmentally immature astrocytes/Müller cells exhibiting a hitherto unknown transient, nonneuronal expression of beta-III tubulin in the context of gliogenesis. Another possibility is that the beta-III tubulin expression in cultured macroglial cells may merely represent a cytoskeletal adaptation of microtubules to cell culture conditions, as previously postulated in retinal epithelial cells [71]. In addition, the findings help to understand the previously described coexpression of GFAP and beta-III tubulin in malignant gliomas [72], probably reflecting a close phenotypic relationship of the latter to NSCs or partially committed glial progenitors. To screen for additional hints of transdifferentiation in retinal glial cells migrating out of the retinal explants, we performed immunohistochemistry with anti-nestin and again found a partial match with GFAP (S1 Fig). Recently, several studies have shown that the expression of nestin is induced in reactive astrocytes of the brain [73] and Müller glial cells and astrocytes of the retina [59]. Nestin is a characterized class IV intermediate filament expressed in the early CNS, first described in the neural tube. It has long been used as a cell distinguishing marker for radial glia and now is increasingly used as a marker for neural progenitor cells. Thus, the induced expression of GFAP and nestin-positive intermediate filaments as well as beta-III tubulin-positive microtubules in some Müller glial cells and astrocytes adjoining regeneration of RGC suggest a potential neuroprotective mechanism in response to IRI.

Our study has limitations worth noting. (a) As the number of cases is still small (n = 7), the results should be judged with care. In the organ culture experiments, differences between IRI+ALF and IRI+iALF did not reach statistical significance, probably due to significant inter- and intraindividual fluctuation of cellular growth out of each retinal explant. Although homogeneity of explant size and RGC content was the goal and intended result of the trepan technique used here, results from the individual explants showed inconsistency. We tried to circumvent this problem by averaging the results from the explants of a single retina in advance. However, quartiles of box plots demonstrated the relevance of this methodological problem also within the treatment groups. The following explanations can be considered: 1. The susceptibility of RGC against IRI differed among the retinal quadrants. 2. Explants cannot be controlled in the quality of attachment to the laminin-1 coated culture ground. 3. This study was underpowered.

(b) At this time, we do not know to which extent ALFs effects on signaling, stress and cell death reported in the first part of this study were responsible for the effects on migration and regeneration in our organ cultures. Future studies using inhibitors and activators or pharmacological studies would begin to answer this.

(c) Interventions in one eye can affect the contralateral eye in different ways [74, 75]. Hence, the contralateral eyes used in this study were not a statistically independent variable. To our best knowledge, the sinistral IRI treatment had no major impact on right sided RGC viability and target molecules analyzed here. This was proven elsewere [17, 21] and seen in Figs 1, 2, 6, 7 and 8, were the data of the contralateral eyes are shown in absolute numbers. However, mRNA results in IRI injured retinal tissue were calculated in relation to the corresponding gene expression in the non-injured retinal tissue of each individual animal (ΔΔCT). This possibly will avoid false-positive results but is a limitation worth noting.

In conclusion, the retina is a dynamic and interconnected network of cells. Most of the current therapies under investigation do not take this integrative nature into account, targeting only one cell type or a single cell process or pathway. CO had been shown to antagonize neurodegeneration and neuroinflammation in the brain and retina by targeting both neurons and the retinal glia in a multimodal approach. The exact mechanisms by which ALF mediated its neuroprotective and neuroregenerative effects after IRI need further analysis, thus opening a new field of CO studies in ophthalmology and possible therapeutic applications.

## Supporting information

S1 Fig GFAP and Nestin coexpression in culture. **(A-D)** Representative images of an IRI+ALF-treated retina in culture showing DAPI nuclear stain (A), GFAP (B), nestin (C), and composite (D). **(E-H)** Representative images of a single cell after IRI+iALF treatment; cell nuclei were stained with DAPI (E), GFAP (F), nestin (G), and composite (H). Scale bar upper row 200 μm, lower row 50 μm.
(TIF)
