# Supplementary figures and images for: Neuroprotection and neuroregeneration of retinal ganglion cells after intravitreal carbon monoxide release
Source: PLoS One. 2017 Nov 27;12(11):e0188444. doi: 10.1371/journal.pone.0188444 (PMC5703485; doi:10.1371/journal.pone.0188444)

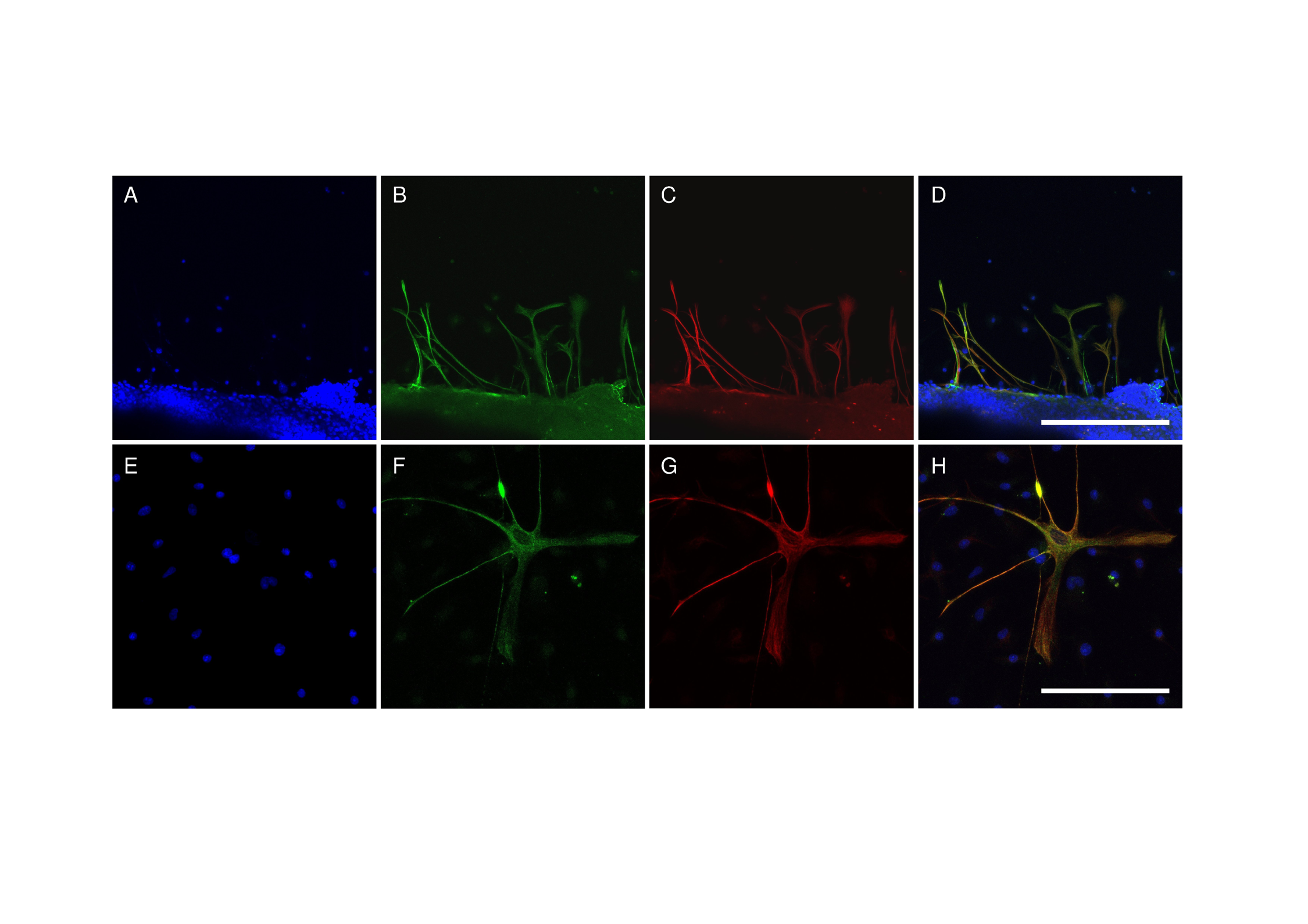

Supplement: S1 Fig — (A-D) Representative images of an IRI+ALF-treated retina in culture showing DAPI nuclear stain (A), GFAP (B), nestin (C), and composite (D). (E-H) Representative images of a single cell after IRI+iALF treatment; cell nuclei were stained with DAPI (E), GFAP (F), nestin (G), and composite (H). Scale bar upper row 200 μm, lower row 50 μm. (TIF) [file pone.0188444.s001.tif]
